# Trends of Antibiotic Resistance Patterns and Bacteriological Profiles of Pathogens Associated with Genitourinary Infections in Secondary Healthcare Facilities in the Volta Region of Ghana

**DOI:** 10.3390/pathogens14070696

**Published:** 2025-07-15

**Authors:** Hayford Odoi, Naodiah Opoku, Brigham Adusei, Kenneth Danquah, Gilbert Vordzogbe, Divine Mayer, Araba Hutton-Nyameaye, Jonathan Jato, Samuel O. Somuah, Emmanuel Orman, Inemesit O. Ben, Thelma A. Aku, Rita Sewornu, Preet Panesar, Yogini H. Jani, Cornelius C. Dodoo

**Affiliations:** 1 School of Pharmacy, University of Health and Allied Sciences, Ho P.O. Box PMB 31, Ghana; hodoi@uhas.edu.gh (H.O.); nopoku20@sop.uhas.edu.gh (N.O.); badusei20@sop.uhas.edu.gh (B.A.); kdanquah20@sop.uhas.edu.gh (K.D.); aharaba@uhas.edu.gh (A.H.-N.); jjato@uhas.edu.gh (J.J.); sosomuah@uhas.edu.gh (S.O.S.); eorman@uhas.edu.gh (E.O.); ioben@uhas.edu.gh (I.O.B.); talalbila@uhas.edu.gh (T.A.A.); 2 Margret Marquart Catholic Hospital, Kpando P.O. Box. 97, Ghana; delavord@hotmail.com; 3 Volta Regional Hospital, Hohoe P.O. Box 27, Ghana; mayerdivineyao24@gmail.com; 4 Ho Teaching Hospital, Ho P.O. Box MA 374, Ghana; ritayaasewornu@gmail.com; 5 Centre for Medicines Optimisation Research and Education, University College London Hospitals NHS Foundation Trust, London NW1 2PG, UK; preet.panesar@nhs.net (P.P.); yogini.jani@nhs.net (Y.H.J.); 6 Research Department of Practice and Policy, School of Pharmacy, University College London, London WC1N 1AX, UK

**Keywords:** genitourinary infections, antimicrobial resistance, antibiogram, secondary healthcare facilities

## Abstract

Urogenital infections contribute greatly to both hospital- and community-acquired infections. In Ghana, the prevalence of resistance to commonly used antibiotics is relatively high. This study sought to evaluate the antibiotic sensitivity of bacterial urogenital pathogens from patient samples in a regional and district hospital in the Volta Region of Ghana. A retrospective cross-sectional study was conducted using data obtained between January and December 2023 from Volta Regional Hospital and Margret Marquart Catholic Hospital. Bacteria were isolated from urine, urethral swabs, and vaginal swabs from 204 patients. Data on culture and sensitivity assays performed using the Kirby–Bauer disc diffusion method were extracted and analyzed using WHONET. The most prevalent organisms isolated from the samples from both facilities were *Escherichia coli* (24.9%), *Staphylococcus aureus* (21.5%), and *Klebsiella oxytoca* (8.8%). The isolates were mostly resistant to amoxicillin/clavulanic acid (n = 75, 95% CI [91.8–99.9]), meropenem (n = 61, 95% CI [87.6–99.4]), cefuroxime (n = 54, 95% CI [78.9–96.5]), ampicillin (n = 124, 95% CI [61.2–77.9]), and piperacillin (n = 43, 95% CI [82.9–99.2]). Multidrug-resistant (MDR, 70 (34.1%)), extensively drug-resistant (XDR, 63 (30.7%)), and pandrug-resistant (PDR, 9 (4.3%)) strains of *S. aureus*, *E. coli,* and *Pseudomonas aeruginosa* were identified from the patient samples. The study highlights the presence of high-priority resistant urogenital pathogens of public health significance to varied antibiotic groups.

## 1. Introduction

The escalating global spread of antimicrobial resistance (AMR) poses a serious threat to public health. In 2015, the World Health Organization (WHO) approved a Global Action Plan to tackle AMR and launched the Global Antimicrobial Resistance and Use Surveillance System (GLASS) [1]. These efforts targeted at curbing AMR promote antimicrobial stewardship, routine, and focused surveillance and the need for surveys and studies that support the timely detection, reporting, risk assessment, and monitoring of emerging resistance.

As bacteria develop resistance to antibiotics, the effectiveness of these essential treatments for the management of infectious diseases declines [2]. The overuse and prolonged use of antibiotics have created major challenges with resistant organisms, leading to higher rates of illness and death [3]. The resistant organisms may be classified as multidrug-resistant (MDR), extensively drug-resistant (XDR), and pandrug-resistant (PDR). MDR organisms are resistant to at least one agent in three or more antimicrobial categories, while XDR strains are resistant to at least one agent in all but two or fewer antimicrobial categories. Strains that are resistant to all antimicrobial agents in all antimicrobial categories are considered PDR [4]. It is estimated that the effectiveness of antibiotics is decreasing, with increasing resistance to both first-line and last-resort antibiotics, resulting in severe clinical, societal, and economic consequences [5]. As a result, drug-resistant pathogens have a profound effect on public health, economic stability, and global mortality rates, with microbial diseases causing a considerable number of deaths annually, according to WHO [6]. The growing prevalence of antibiotic-resistant bacteria is a major concern for both inpatient and outpatient care, especially given the slow development of new antimicrobials [7]. Pathogens such as *Enterococcus faecium*, *Klebsiella pneumoniae*, *Escherichia coli*, *Pseudomonas aeruginosa*, and *Enterobacter* spp. are closely monitored due to their resistance to various antibacterial agents [8,9].

Surveillance of resistance in bacterial species is core to overcoming the burden of AMR [10]. The selection of antibacterials for empiric and definitive therapy relies on well-curated data on the resistance profiles of bacterial co-infections in various diseases. Urogenital infections are made up of a spectrum of urinary tract-related infections, from asymptomatic bacteriuria and cystitis to pyelonephritis and urosepsis [11]. Urinary tract infections (UTI) are among the most common nosocomial and community-acquired infections affecting both the elderly and adults of reproductive age [12]. These infections are associated with several risk factors like comorbid diseases, functional status, and living environments. Evaluation of the antibiotic susceptibility profiles of urogenital pathogens has been associated with increased antibiotic efficacy and appropriate antibiotic selection for management of these genitourinary infections.

An antibiogram is an important resource in healthcare, offering a periodic overview of the antimicrobial susceptibility of bacterial isolates [13,14]. Clinicians use antibiograms to evaluate local susceptibility rates, compare these rates with other institutions, guide the selection of empiric antibiotic treatments, and track resistance trends over time within their facility. Therefore, the aim of this work was to assess the antibiotic susceptibility profiles of urogenital pathogens isolated from urogenital infections in secondary healthcare facilities in the Volta Region of Ghana.

## 2. Materials and Methods

### 2.1. Study Site

**Figure 1:**
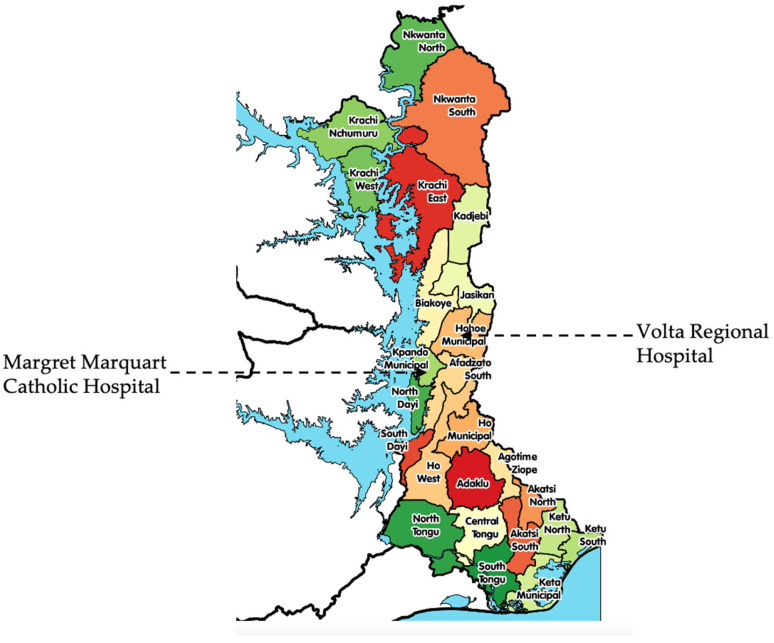
A map of the Volta Region showing the location of the study sites.

This study was carried out at the Volta Regional Hospital (VRH), Hohoe, and the Margret Marquart Catholic Hospital (MMCH), Kpando (Figure 1). These hospitals are secondary healthcare facilities that serve the health needs of patients in the upper belt of the Volta Region of Ghana. These facilities together have more than 300 beds and functional microbiology laboratories that conduct routine isolation and characterization of bacteria co-infecting varied disease conditions.

### 2.2. Study Design

This was a retrospective cross-sectional study utilizing reports from the Lightwave Health Information Management System and the laboratory data register on all isolated urogenital micro-organisms in VRH and MMCH. Laboratory data on the culture and sensitivity of bacterial isolates from January 2023 to December 2023 were assessed. The microbiology laboratory receives urogenital specimens, including vaginal swabs, urine, and urethral swabs. The bacteriological analysis of these samples included enrichment and culturing of pathogens of interest employing national standard operating procedures and guidelines from the 33rd edition of the performance standards for antimicrobial susceptibility testing by the Clinical and Laboratory Standards Institute (CLSI, 2023 [15]). Bacteria were enriched in blood agar. Routine biochemical assays were performed using triple-sugar iron broth, lysine iron agar, urea media, oxidase reagent, and analytical profile index (API) 20E for Enterobacterales; these were all obtained from Oxoid Limited (Basingstoke, UK). Quality control was performed with standard microbial strains, such as *Staphylococcus aureus* (ATCC 6538), *Escherichia coli* (ATCC 8739), *Pseudomonas aeruginosa* (ATCC 27853), *Proteus mirabilis* (ATCC 12453), *Haemophilus influenza* (ATCC 9027), and *Enterococcus faecalis* (ATCC 29212), and culture and sensitivity testing was performed using the Kirby–Bauer disc diffusion method employing CLSI breakpoints [15].

### 2.3. Inclusion Criteria and Exclusion Criteria

All urogenital microbiological specimen entries from January 2023 to December 2023 with information on patient age, gender, ward, outpatient/inpatient status, specimen type, and antibiotic culture and sensitivity results of bacterial isolates implicated in suspected infections were included in the study. The data set excluded viral strains, fungal strains, and other infectious protozoal pathogens.

### 2.4. Statistical Analysis

Microsoft Excel (Version 16), Origin Pro (2025), and WHONET software (2024), developed by the WHO Collaborating Centre for Surveillance of Antimicrobial Resistance, were used to analyze the data. Data were analyzed using tables, graphs, and charts. The prevalence of the isolates from the samples was calculated by analysis of isolate frequency per patient. The percentage susceptibility of the isolates was also determined at a 95% confidence interval for each isolate type of infectious pathogen isolated. A clustering heatmap using group average as a clustering method was used to assess the similarity in antibiotic response between the urogenital pathogens.

## 3. Results

### 3.1. Distribution of Bacterial Isolates in Characteristic Patient Groups

A total of 205 specimens from 204 patients with positive culture tests obtained from both facilities met the inclusion criteria and were included in the study (Table 1). Twenty-five distinct bacterial species belonging to 12 bacterial genera were identified in the samples, of which 11 were Gram-positive while 14 were Gram-negative. *Staphylococcus aureus* (n = 44, 21.5%) and *Escherichia coli* (n = 51, 24.9%) were the most prevalent bacterial isolates from both hospital facilities. Samples from which positive bacterial cultures were obtained from both facilities were urine (n = 105), vaginal (n = 87), and urethral swabs (n = 13). *S. aureus* was the most isolated organism from urethral and vaginal samples. *E. coli* and *Klebsiella oxytoca* were more prevalent in the urine samples from both study facilities. *Pseudomonas aeruginosa* was identified in urine samples from five patients. *Neisseria gonorrhoeae* was found in urethral swabs (n = 1) and vaginal swabs (n = 2).

**Figure 2:**
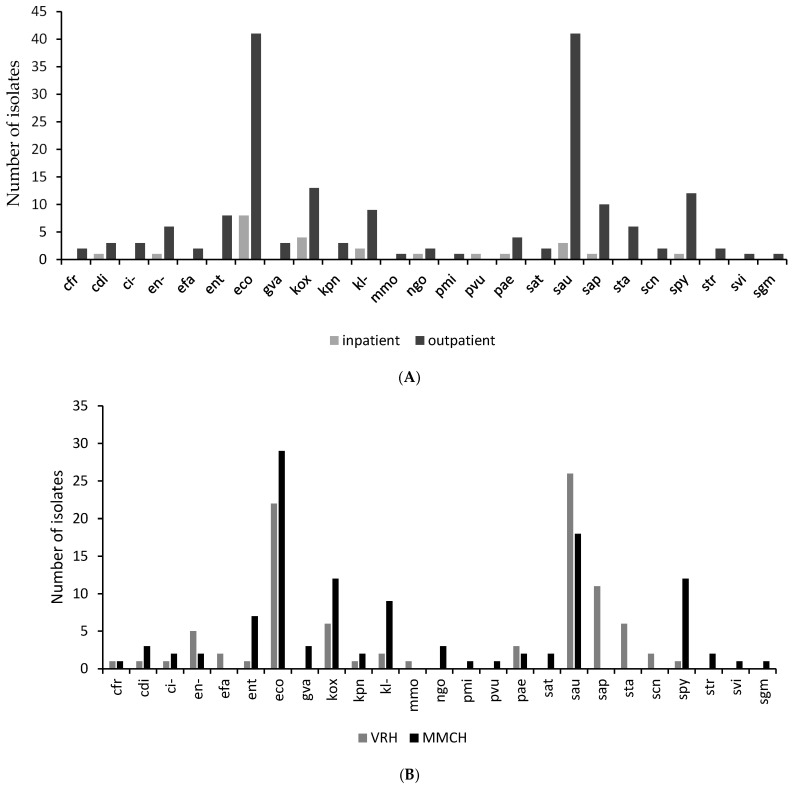
A graph showing the organism distribution by comparing the number of isolates: (**A**) by department (i.e., Inpatient and Outpatient Departments) in the study sites; (**B**) by facility (i.e., Margret Marquart Catholic Hospital and Volta Regional Hospital). *Escherichia coli* (eco); *Pseudomonas aeruginosa* (pae); *Klebsiella* sp. (kl-); *Citrobacter* sp. (ci-); *Klebsiella oxytoca* (kox); *Staphylococcus aureus* ss. aureus (sau) *Citrobacter koseri* (cdi); *Enterococcus* sp. (ent); *Proteus vulgaris* (pvu); *Klebsiella pneumoniae* ss. *pneumoniae* (kpn); *Proteus mirabilis* (pmi); *Staphylococcus saprophyticus* ss. saprophyticus (sap); *Citrobacter freundii* (cfr); *Enterobacter* sp. (en-); *Morganella morganii* ss. Morganii (mmo); *Salmonella typhi* (sat); *Streptococcus viridans*, alpha-hem. (svi); *Staphylococcus*, coagulase-negative (scn); *Streptococcus pyogenes* (spy); *Staphylococcus* spp. (sta); *Gardnerella vaginalis* (gva); *Streptococcus* spp. (str); *Enterococcus faecalis* (efa); *Streptococcus*, *non-haemolytic* (*gamma*) (sgm).

There was a higher prevalence of urogenital pathogens in outpatient (88.3%) compared to inpatient urogenital samples. The isolates from VRH and the MMCH each showed a similar distribution to the total sample distribution from both facilities. *Escherichia coli* was the most isolated organism from the MMCH, while *Staphylococcus aureus* was the most predominant isolate from samples from the VRH (Figure 2A). *Klebsiella oxytoca*, *Staphylococcus saprophyticus*, *Klebsiella* sp., *Streptococcus pyogenes*, and *Pseudomonas aeruginosa* were also common in inpatient samples from both facilities (Figure 2B).

**Figure 3:**
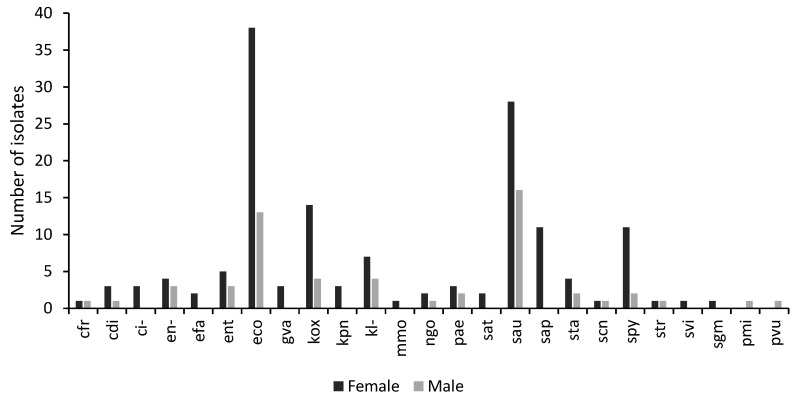
A graph showing the distribution of isolates by gender. The number of isolates per microorganism obtained from samples taken from males and females visiting the study sites with genitourinary infections. *Escherichia coli* (eco); *Pseudomonas aeruginosa* (pae); *Klebsiella* sp. (kl-); *Citrobacter* sp. (ci-); *Klebsiella oxytoca* (kox); *Staphylococcus aureus* ss. aureus (sau) *Citrobacter koseri* (cdi); *Enterococcus* sp. (ent); *Proteus vulgaris* (pvu); *Klebsiella pneumoniae* ss. *pneumoniae* (kpn); *Proteus mirabilis* (pmi); *Staphylococcus saprophyticus* ss. saprophyticus (sap); *Citrobacter freundii* (cfr); *Enterobacter* sp. (en-); *Morganella morganii* ss. Morganii (mmo); *Salmonella typhi* (sat); *Streptococcus viridans*, alpha-hem. (svi); *Staphylococcus*, coagulase-negative (scn); *Streptococcus pyogenes* (spy); *Staphylococcus* spp. (sta); *Gardnerella vaginalis* (gva); *Streptococcus* spp. (str); *Enterococcus faecalis* (efa); *Streptococcus*, *non-haemolytic* (*gamma*) (sgm).

About 73% of the samples were obtained from females, while 27% of the samples were from males. Bacterial isolates were predominantly obtained from patients between the ages of 25 and 34 years for both genders. Female samples had a higher prevalence of *E. coli*, *Klebsiella oxytoca*, *Staphylococcus aureus*, *Staphylococcus saprophyticus*, and *Streptococcus pyogenes* compared to the samples obtained from males (Figure 3).

### 3.2. The Antibiotic Resistance Patterns of Bacterial Isolates

The isolated urogenital pathogens were tested against a total of 21 antibiotics using a standardized Kirby–Bauer disc diffusion assay according to guidelines from the 33rd edition (2023 version) of the performance standards for antimicrobial susceptibility testing of the Clinical and Laboratory Standards Institute (CLSI). The results, however, excluded organisms that are intrinsically resistant to the tested antibiotics [16]. Overall, the isolates were most resistant to amoxicillin/clavulanic acid (n = 75, 95% CI [91.8–99.9]), meropenem (n = 61, 95% CI [87.6–99.4]), cefuroxime (n = 54, 95%CI [ 78.9–96.5]), and piperacillin (n = 43, 95% CI [82.9–99.2]). Amikacin (n = 135, 95% CI [5.4–16.2], gentamicin (n = 146, 95% CI [22.4–37.7]), levofloxacin (n = 200, 95% CI [24.2–40.2], and cefotaxime (n = 68, 40% CI [27–50.9] were the most active antibiotics against all the bacterial isolates from both facilities.

Ciprofloxacin (n = 200), tetracycline (n = 185), gentamicin (n = 146), and trimethoprim/sulfamethoxazole (n = 151) were the antibiotics that were frequently tested against the isolated pathogens. Piperacillin, ampicillin, ampicillin/sulbactam, amikacin, nitrofurantoin, ciprofloxacin, and erythromycin all showed greater activity against test isolates from the VRH compared to the MMCH. Levofloxacin, chloramphenicol, and vancomycin were more active against isolates from MMCH compared to VRH.

Of the 21 antibiotics tested, amikacin was the most active against *E. coli*, *K. oxytoca*, *S. aureus*, and *P. aeruginosa* isolates from both hospitals. Gentamicin showed the highest activity (n = 91%) against *Streptococcus pyogenes*, *S. aureus*, and *E. coli* isolates had no sensitivity to ampicillin/sulbactam and amoxicillin/clavulanic acid. Norfloxacin and chloramphenicol also showed no activity against the *S. aureus* isolates (Table S1). Piperacillin, amoxicillin/clavulanic acid, and tetracycline were not active against the *E. coli* isolates. Meropenem was also not active against any of the *P. aeruginosa* isolates. Meropenem also showed no activity against all the isolated urogenital pathogens except *E. coli.*

**Figure 4:**
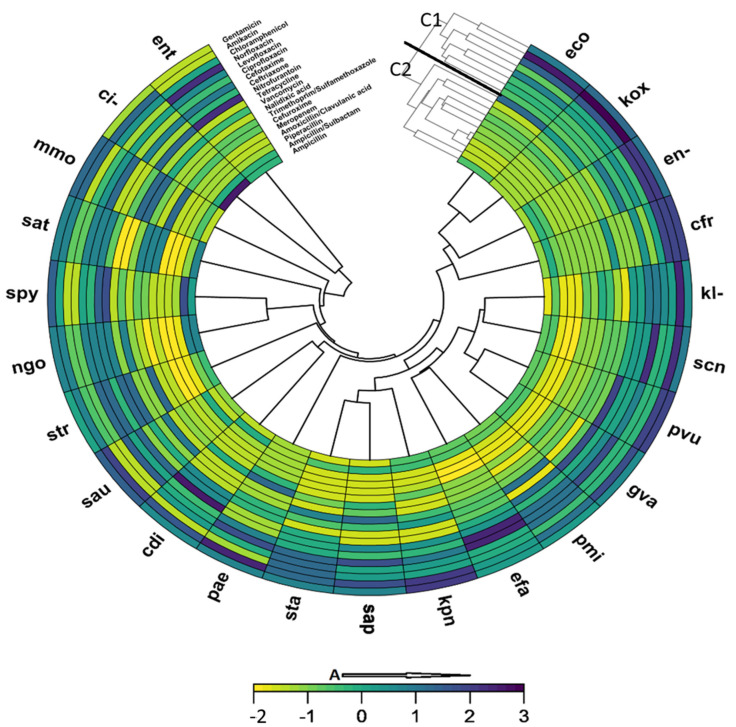
A clustering heatmap showing similarity in antibiotic susceptibility of isolated urogenital pathogens: A: Increasing susceptibility; C1: Group 1 cluster; C2: Group 2 cluster. The most active antibiotics are clustered in group one (C1), while the second cluster (C2) contains antibiotics that were less effective against the urogenital pathogens. (ci-); *Klebsiella oxytoca* (kox); *Staphylococcus aureus* (sau); *Citrobacter koseri* (cdi); *Acinetobacter* sp. (ac-); *Enterococcus* sp. (ent); *Proteus vulgaris* (pvu); *Klebsiella pneumoniae* (kpn); *Proteus mirabilis* (pmi); *Staphylococcus saprophyticus* ss. Saprophyticus (sap); *Staphylococcus epidermidis* (sep); *Providencia* sp. (prv); *Citrobacter freundii* (cfr); *Serratia marcescens* (sma); *Enterobacter* sp. (en-); *Moraxella catarrhalis* (bca); *Morganella morganii* ss. Morganii (mmo); *Shigella* sp. (shi); *Providencia rettgeri* (pre); *Salmonella* sp. (sal); *Salmonella typhi* (sat); *Streptococcus viridans*, alpha-hem. (svi); *Francisella tularensis* ss. Tularensis (ftu); *Klebsiella pneumoniae* ss. Rhinoscleromatis (krn); *Neisseria meningitidis* (nme); *Staphylococcus*, coagulase-negative (scn); *Streptococcus pyogenes* (spy).

The activity of the antibiotics against the isolated urogenital pathogens was clustered into two groups (Figure 4). Gentamicin, amikacin, chloramphenicol, norfloxacin, levofloxacin, ciprofloxacin, cefotaxime, and ceftriaxone were distributed into the group one cluster, while the other antibiotics were also distributed into a separate cluster. At a similarity of 98%, meropenem and amoxicillin showed similar activity profiles against the isolated urogenital pathogens. The most active antibiotics clustered in group one, while the second cluster contained antibiotics that were less effective against the urogenital pathogens. There was about 50% similarity in the sensitivity profiles of *E. coli* and *K. oxytoca* to the antibiotics tested.

A significant prevalence of MDR, XDR, and PDR strains of *S. aureus*, *E. coli*, and *P. aeruginosa* was observed in the clinical specimens. A total of 70 MDR, 63 XDR, and 9 PDR strains were identified; 75% (n = 38) and 65% (n = 33) of the *E. coli* isolates were MDR and XDR, respectively, while one PDR strain was isolated. One PDR strain of P. aeruginosa was also isolated from the urogenital samples. Of the total *S. aureus* isolates, 66% were MDR, while 61% and 16% were XDR and PDR strains, respectively. Additionally, a striking 65% of *Klebsiella* spp. were identified as XDR.

The data also indicated a high incidence of extended-spectrum β-lactamase (ESBL)-producing (n = 68) Enterobacterales, carbapenem-resistant Enterobacterales (CRE) (n = 19), and vancomycin-resistant *Staphylococcus* spp. (Table S2).

## 4. Discussion

Surveillance plays a critical role in antimicrobial stewardship by providing essential data that informs and guides effective interventions and policies to combat AMR. Surveillance efforts help in epidemiological data collection, identification of resistance patterns, and outbreak detection. These efforts inform appropriate selection of antimicrobials for empiric and definitive therapy [17]. Global efforts made by networks such as the WHO GLASS, Africa Centers for Disease Control and Prevention’s AMR surveillance network, and other initiatives by partner organizations have a primary focus on pathogen surveillance and characterization. The use of bacteria prevalence studies in the clinical setting is indispensable in managing bacterial infections and safeguarding the efficacy of antibiotics. Clinicians make better choices for empiric therapy when local bacterial prevalence studies are available. According to Cressman and colleagues, empiric antibiotic treatment should effectively cover approximately 80% of expected bacteria in non-critically ill patients and 90% in critically ill patients [18].

Urogenital infections form a major part of nosocomial and community-acquired infections [19]. Urogenital pathogens are associated with generalized UTI, cystitis, gonorrhea, syphilis, chlamydiosis, and trichomoniasis. In most secondary healthcare facilities in Ghana, antibiotic selection for the management of UTI is empiric [20]. The study thus focused on assessing the trend of antibiotic resistance in bacterial urogenital pathogens from district-level facilities in the Volta region of Ghana.

Findings from this study revealed a high proportion of isolates from female patients. This could be because women have a better health-seeking behaviour, hence resulting in thorough bacteriological screening of suspected infections during hospital visits [21]. These findings are comparable to those reported by Ahabwe and colleagues, who also isolated a substantial proportion (62%) of bacterial isolates from women. Women are also more prone to UTIs due to their anatomical characteristics [22,23]. This may have accounted for the high number of urogenital pathogens in the samples from both hospitals. Most of the isolates were also obtained from patients predominantly in their reproductive age range, likely due to reproductive-age-related urinary tract infections like candidiasis, gonorrhea, chlamydia, syphilis, and trichomoniasis, which may require regular hospital visits and screening [24,25]. Bacterial vaginosis, gonorrhea, pelvic inflammatory disease, and UTI are associated with urogenital pathogens such as *Escherichia coli*, *Proteus mirabilis*, *Klebsiella pneumoniae*, *Staphylococcus saprophyticus*, and *Neisseria gonorrhoeae*, and these urogenital pathogens were also prevalent in the urogenital samples from both facilities [26,27,28,29].

The source clinical samples were urine, urethral swabs, and vaginal swabs. Urine and vaginal swab isolates were the most predominant. This indicates the presentation of cases of both symptomatic and asymptomatic bacteriuria, urethritis, and vaginosis in both hospitals. Globally, UTIs represent almost 10% of all infection cases in hospitalized patients, which makes them the second most common cause of emergency admission, and can infect various parts of the urinary system, including the bladder (cystitis), kidneys (pyelonephritis), and urethra (urethritis) [19,30,31]. *Escherichia coli*, *Staphylococcus aureus*, and *Klebsiella oxytoca* were the most common bacterial isolates from urine. Though *S. aureus* is less commonly isolated from urine samples compared to *E. coli*, its presence is more commonly seen in patients with underlying health conditions such as diabetes or those who are immunocompromised [11,32]. *Streptococcus pyogenes* was also isolated from vaginal samples. Though this pathogen frequently colonizes the respiratory tract, it can occasionally colonize the vaginal tract when there is dysbiosis in the vaginal flora [25,28,33].

Antibacterials account for 73–93% of all anti-infectives used by hospital facilities in the Volta Region [34]. In the current study, urogenital pathogenic bacterial isolates from the various sample types were resistant to most of the commonly used antibacterials. The resistance of the pathogens to β-lactam antibacterials such as penicillins, carbapenems, and cephalosporins is particularly worrying since these antibacterials form a major part of the routine prescribing of antibacterials for empiric therapy in the study region. These findings are consistent with antibacterial susceptibility data emerging from the major referral center in the same region and other hospital facilities in Ghana [25,35,36,37].

Most antibiotics in the ‘Access’ group of the WHO AWaRe classification were also found to be less potent against most of the pathogens targeted [38]. However, amikacin and gentamicin showed a high potency against both Gram-positive and Gram-negative isolates from both hospitals. The ‘Watch’ category of antibacterials, such as piperacillin, meropenem, cefuroxime, and vancomycin, showed low activity against the study bacterial strains. This is particularly worrying since these antibacterials are less frequently used in these hospitals. Resistance to these less readily available and used antibacterials may be caused by cross-resistance of antibiotic resistance determinants such as plasmids, transposons, and integrons among antibacterials of the same class [39,40]. Amikacin, gentamicin, and levofloxacin were the most effective antibiotics against the urogenital pathogenic bacterial isolates. The low resistance of common pathogens to these antibacterials has been highlighted by various studies across the African sub-region [10].

There was a high prevalence of ESBL-producing Enterobacterales (n = 68), carbapenem-resistant Enterobacterales (CRE), and vancomycin-resistant staphylococci. Extended-spectrum β-lactamase-producing Enterobacterales (ESBL-E) poses a significant global health challenge as it frequently causes the failure of empirical antibiotic therapy. The high resistance in these pathogens may be mediated by antibiotic resistance genotypes such as CTX-M, TEM, and SHV variants [41]. The *E. coli* and *K. pneumoniae*-derived CTX-M genotype is one of the major types of ESBLs. These may be disseminated by mobile genetic elements among the bacterial population. Carbapenems are considered the antibiotics of choice for the treatment of serious infections caused by ceftriaxone-resistant Enterobacterales [41]. This surge in CRE is mostly driven by the emergence and spread of variants of carbapenemases such as metallo-β-lactamase IMP, VIM, and KPC type variants [42]. The occurrence of MDR, XDR, and PDR strains may be due to inappropriate and indiscriminate use of antibiotics and horizontal transfer of resistance genes in bacteria [43]. These resistant strains have significant public health implications, and further studies are therefore needed to establish the cause and impact of this occurrence in the study hospitals.

The data presented in this study are fundamental to antimicrobial resistance surveillance at the district level in the study region. However, there are pertinent limitations that need to be addressed. Both facilities should make efforts to isolate and test pathogens against all the routinely used antibacterials. This is because some bacterial isolates were tested only against a few antimicrobials, which may skew the resistance data. Also, the facilities should ensure samples are collected before antibiotic therapy since prior antibiotic treatment before sample collection may over-stress the overall antimicrobial resistance outlook for the facility. Another limitation was that some of the bacterial isolates were not identified at the species level due to resource limitations in both facilities. The resource limitations restricted pathogen identification during routine culture and sensitivity testing to the use of biochemical assays. Also, the reliance on antibiotic multidiscs for routine culture and sensitivity tests may lead to testing the sensitivity of isolates to antibiotics that they are intrinsically resistant to.

## 5. Conclusions

The findings from the study indicated a high prevalence of *E. coli* and *S. aureus* in the study samples. Most of the isolates from these samples showed great susceptibility to amikacin, gentamicin, and levofloxacin, while high resistance was observed in the β-lactam group of antibiotics. The high prevalence of WHO priority organisms such as vancomycin-resistant Enterococcus, ESBL-E, and CRE highlights the need for both facilities to be zoned in as a hotspot for AMR surveillance efforts.

Given that AMR is a worldwide issue that calls for international cooperation to solve, it is critical that AMR surveillance data be produced in order to give stakeholders and healthcare professionals some information to aid in decision-making. Particular difficulties exist in sub-Saharan Africa, where the majority of medical facilities lack enough funding, and some patients cannot afford even the most basic culture and sensitivity tests. Ministries of health should, however, make an effort to cover a portion of these expenses in order to eliminate or lower patient costs. Incorporating molecular diagnostics into these tests will also be crucial for improved pathogen and resistance identification.

## Figures and Tables

**Table 1:** Prevalence of bacterial urogenital pathogens in samples from the Volta Regional Hospital and the Margret Marquart Catholic Hospital (N = 205).

	Organism	Code	Urethral Swab	Urine	HVS Swab	Number of Isolates	(%)
Gram-negative	*Escherichia coli*	eco		37	14	51	24.9
	*Klebsiella oxytoca*	kox		14	4	18	8.8
	*Klebsiella* sp. *	kl-		7	4	11	5.4
	*Enterobacter* sp. *	en-		4	3	7	3.4
	*Pseudomonas aeruginosa*	pae		5		5	2.4
	*Citrobacter koseri*	cdi		2	2	4	2
	*Citrobacter* sp. *	ci-		3		3	1.5
	*Klebsiella pneumoniae*	kpn		3		3	1.5
	*Neisseria gonorrhoeae*	ngo	1		2	3	1.5
	*Citrobacter freundii*	cfr		1	1	2	1
	*Salmonella Typhi*	sat		1	1	2	1
	*Morganella morganii*	mmo			1	1	0.5
	*Proteus mirabilis*	pmi		1		1	0.5
	*Proteus vulgaris*	pvu		1		1	0.5
Gram-positive	*Staphylococcus aureus*	sau	10	10	24	44	21.5
	*Streptococcus pyogenes*	spy	1	1	11	13	6.3
	*Staphylococcus saprophyticus*	sap		5	6	11	5.4
	*Enterococcus* sp. *	ent		5	3	8	3.9
	*Staphylococcus* sp. *	sta	1	1	4	6	2.9
	*Gardnerella vaginalis*	gva			3	3	1.5
	*Staphylococcus*, coagulase-negative	scn		2		2	1
	*Streptococcus* sp. *	str		1	1	2	1
	*Enterococcus faecalis*	efa		1	1	2	1
	*Streptococcus viridans*, *alpha-hem.*	svi		1		1	0.5
	*Streptococcus*, *non-haemolytic* (*gamma*)	sgm		1		1	0.5

* Other unspecified genera, HVS—high vaginal swabs.

## Data Availability

All data generated or analyzed during this study have been included in this article.

## Supplementary Materials

The following supporting information can be downloaded at: https://www.mdpi.com/article/10.3390/pathogens14070696/s1, Table S1: Resistance pattern of pathogens isolated from sample types from both facilities; Table S2: Public health alerts—Important species and resistance of isolates (WHO Global Priority List of Antibiotic-Resistant Bacteria).

## Abbreviations

The following abbreviations are used in this manuscript:

CLSI	Clinical and Laboratory Standards Institute
CRE	carbapenem-resistant Enterobacterales
ESBL	extended-spectrum β-lactamase
GLASS	Global Antimicrobial Resistance and Use Surveillance System
MDR	Multidrug resistant
MMCH	Margret Marquart Catholic Hospital
PDR	Pandrug resistant
UTI	Urinary tract infections
VRH	Volta Regional Hospital
WHO	World Health Organization
XDR	Extensively drug-resistant
