# Supplementary material for: Trends of Antibiotic Resistance Patterns and Bacteriological Profiles of Pathogens Associated with Genitourinary Infections in Secondary Healthcare Facilities in the Volta Region of Ghana
Source: Pathogens. 2025 Jul 15;14(7):696. doi: 10.3390/pathogens14070696 (PMC12300375; doi:10.3390/pathogens14070696)
Supplement: Supplementary file 1 [file pathogens-14-00696-s001.zip › pathogens-3689725-supplementary.pdf]

Supplementary data

Table S1: Resistance pattern of pathogens isolated from sample types from both facilities.

Organisms	Antibiotic	Number	%R	%R 95%C.I.
Escherichia coli	Ampicillin	28	67.9	47.6-83.4
	Piperacillin	13	100.0	71.7-100
	Amoxicillin/Clavulanic acid	13	100.0	71.7-100
	Ampicillin/Sulbactam	2	100.0	19.8-100
	Cefuroxime	12	91.7	59.8-99.6
	Ceftriaxone	43	69.8	53.7-82.3
	Cefotaxime	15	73.3	44.8-91.1
	Meropenem	12	91.7	59.8-99.6
	Amikacin	49	10.2	3.8-23.0
	Gentamicin	28	46.4	28.0-65.8
	Nalidixic acid	27	85.2	65.4-95.1
	Ciprofloxacin	49	73.5	58.7-84.6
	Levofloxacin	37	32.4	18.6-49.9
	Norfloxacin	20	80.0	55.7-93.4
	Trimethoprim/Sulfamethoxazole	33	78.8	60.6-90.4
	Nitrofurantoin	36	30.6	16.9-48.3
	Chloramphenicol	13	30.8	10.4-61.1
	Tetracycline	45	97.8	86.8-99.9
Klebsiella oxytoca	Piperacillin	5	100.0	46.3-100
	Amoxicillin/Clavulanic acid	5	100.0	46.3-100
	Cefuroxime	2	50.0	2.7-97.3
	Ceftriaxone	18	77.8	51.9-92.6
	Cefotaxime	5	40.0	7.3-83.0
	Meropenem	3	66.7	12.5-98.2
	Amikacin	18	0.0	0.0-21.9
	Gentamicin	10	30.0	8.1-64.6
	Nalidixic acid	12	83.3	50.9-97.1
	Ciprofloxacin	18	77.8	51.9-92.6
	Levofloxacin	16	56.3	30.6-79.2
	Norfloxacin	7	57.1	20.2-88.2
	Trimethoprim/Sulfamethoxazole	13	92.3	62.1-99.6
	Nitrofurantoin	14	50.0	24.0-76.0
	Chloramphenicol	3	33.3	1.8-87.5
	Tetracycline	15	80.0	51.4-94.7
Klebsiella sp.	Piperacillin	2	100.0	19.8-100
	Amoxicillin/Clavulanic acid	2	100.0	19.8-100
	Ceftriaxone	9	77.8	40.2-96.1

	Cefotaxime	5	20.0	1.1-70.1
	Amikacin	10	10.0	0.5-45.9
	Gentamicin	8	25.0	4.5-64.4
	Nalidixic acid	6	66.7	24.1-94.0
	Ciprofloxacin	11	45.5	18.1-75.4
	Levofloxacin	11	27.3	7.3-60.7
	Norfloxacin	2	50.0	2.7-97.3
	Trimethoprim/Sulfamethoxazole	7	85.7	42.0-99.2
	Nitrofurantoin	6	83.3	36.5-99.1
	Tetracycline	10	70.0	35.4-91.9
Enterobacter sp.	Ampicillin	3	66.7	12.5-98.2
	Piperacillin	5	80.0	29.9-98.9
	Amoxicillin/Clavulanic acid	4	100.0	39.6-100
	Cefuroxime	1	0.0	0.0-94.5
	Ceftriaxone	7	71.4	30.3-94.9
	Cefotaxime	3	33.3	1.8-87.5
	Meropenem	1	100.0	5.5-100
	Amikacin	7	0.0	0.0-43.9
	Gentamicin	7	28.6	5.1-69.7
	Nalidixic acid	4	100.0	39.6-100
	Ciprofloxacin	7	57.1	20.2-88.2
	Levofloxacin	6	33.3	6.0-75.9
	Norfloxacin	4	75.0	21.9-98.7
	Trimethoprim/Sulfamethoxazole	3	66.7	12.5-98.2
	Nitrofurantoin	4	100.0	39.6-100
	Chloramphenicol	1	0.0	0.0-94.5
	Tetracycline	7	85.7	42.0-99.2
Pseudomonas aeruginosa				
	Piperacillin	4	100.0	39.6-100
	Meropenem	1	100.0	5.5-100
	Amikacin	4	25.0	1.3-78.1
	Ciprofloxacin	5	60.0	17.0-92.7
	Levofloxacin	5	40.0	7.3-83.0
	Nitrofurantoin	5	80.0	29.9-98.9
Citrobacter koseri	Ampicillin	4	100.0	39.6-100
	Cefuroxime	1	100.0	5.5-100
	Ceftriaxone	4	50.0	9.2-90.8
	Cefotaxime	2	0.0	0.0-80.2
	Meropenem	1	100.0	5.5-100
	Amikacin	4	0.0	0.0-60.4
	Gentamicin	2	50.0	2.7-97.3
	Nalidixic acid	2	100.0	19.8-100

	Ciprofloxacin	4	75.0	21.9-98.7
	Levofloxacin	3	33.3	1.8-87.5
	Trimethoprim/Sulfamethoxazole	3	66.7	12.5-98.2
	Nitrofurantoin	2	50.0	2.7-97.3
	Chloramphenicol	1	100.0	5.5-100
	Tetracycline	3	100.0	31.0-100
Citrobacter sp.	Ampicillin	1	0.0	0.0-94.5
	Piperacillin	1	100.0	5.5-100
	Amoxicillin/Clavulanic acid	1	100.0	5.5-100
	Ceftriaxone	3	100.0	31.0-100
	Amikacin	3	33.3	1.8-87.5
	Gentamicin	1	100.0	5.5-100
	Nalidixic acid	2	100.0	19.8-100
	Ciprofloxacin	3	100.0	31.0-100
	Levofloxacin	3	66.7	12.5-98.2
	Norfloxacin	2	100.0	19.8-100
	Trimethoprim/Sulfamethoxazole	1	100.0	5.5-100
	Nitrofurantoin	3	33.3	1.8-87.5
	Tetracycline	3	100.0	31.0-100
Klebsiella pneumoniae	Piperacillin	1	100.0	5.5-100
	Amoxicillin/Clavulanic acid	1	100.0	5.5-100
	Ceftriaxone	3	100.0	31.0-100
	Amikacin	3	0.0	0.0-69.0
	Gentamicin	1	0.0	0.0-94.5
	Nalidixic acid	2	100.0	19.8-100
	Ciprofloxacin	3	66.7	12.5-98.2
	Levofloxacin	3	0.0	0.0-69.0
	Norfloxacin	2	50.0	2.7-97.3
	Trimethoprim/Sulfamethoxazole	2	100.0	19.8-100
	Nitrofurantoin	3	100.0	31.0-100
	Tetracycline	2	50.0	2.7-97.3
Neisseria gonorrhoeae	Ceftriaxone	3	0.0	0.0-69.0
	Cefotaxime	2	0.0	0.0-80.2
	Amikacin	2	0.0	0.0-80.2
	Ciprofloxacin	3	0.0	0.0-69.0
	Tetracycline	3	0.0	0.0-69.0
Citrobacter freundii	Ampicillin	2	100.0	19.8-100
	Cefuroxime	1	100.0	5.5-100
	Ceftriaxone	2	50.0	2.7-97.3
	Cefotaxime	1	100.0	5.5-100
	Meropenem	1	100.0	5.5-100
	Amikacin	2	0.0	0.0-80.2

	Gentamicin	1	0.0	0.0-94.5
	Nalidixic acid	1	100.0	5.5-100
	Ciprofloxacin	2	50.0	2.7-97.3
	Levofloxacin	1	100.0	5.5-100
	Trimethoprim/Sulfamethoxazole	2	100.0	19.8-100
	Nitrofurantoin	1	100.0	5.5-100
	Chloramphenicol	1	0.0	0.0-94.5
	Tetracycline	2	50.0	2.7-97.3
Salmonella Typhi	Ampicillin	1	0.0	0.0-94.5
	Ceftriaxone	2	0.0	0.0-80.2
	Cefotaxime	1	0.0	0.0-94.5
	Ciprofloxacin	2	0.0	0.0-80.2
	Tetracycline	2	50.0	2.7-97.3
Morganella morganii	Ampicillin	1	100.0	5.5-100
	Cefuroxime	1	0.0	0.0-94.5
	Ceftriaxone	1	0.0	0.0-94.5
	Cefotaxime	1	100.0	5.5-100
	Meropenem	1	100.0	5.5-100
	Amikacin	1	0.0	0.0-94.5
	Gentamicin	1	0.0	0.0-94.5
	Ciprofloxacin	1	0.0	0.0-94.5
	Trimethoprim/Sulfamethoxazole	1	100.0	5.5-100
	Chloramphenicol	1	100.0	5.5-100
	Tetracycline	1	0.0	0.0-94.5
Proteus mirabilis	Ceftriaxone	1	0.0	0.0-94.5
	Amikacin	1	0.0	0.0-94.5
	Ciprofloxacin	1	0.0	0.0-94.5
	Levofloxacin	1	0.0	0.0-94.5
	Norfloxacin	1	0.0	0.0-94.5
	Trimethoprim/Sulfamethoxazole	1	0.0	0.0-94.5
	Nitrofurantoin	1	100.0	5.5-100
	Tetracycline	1	0.0	0.0-94.5
Proteus vulgaris	Ampicillin/Sulbactam	1	100.0	5.5-100
	Ceftriaxone	1	0.0	0.0-94.5
	Cefotaxime	1	0.0	0.0-94.5
	Meropenem	1	100.0	5.5-100
	Amikacin	1	0.0	0.0-94.5
	Gentamicin	1	0.0	0.0-94.5
	Ciprofloxacin	1	100.0	5.5-100
	Levofloxacin	1	100.0	5.5-100
	Norfloxacin	1	100.0	5.5-100
	Trimethoprim/Sulfamethoxazole	1	100.0	5.5-100

	Nitrofurantoin	1	100.0	5.5-100
	Chloramphenicol	1	100.0	5.5-100
	Tetracycline	1	100.0	5.5-100
Staphylococcus aureus	Penicillin G	23	73.9	51.3-88.9
	Ampicillin	33	75.8	57.4-88.3
	Amoxicillin/Clavulanic acid	25	96.0	77.7-99.8
	Ampicillin/Sulbactam	4	75.0	21.9-98.7
	Cefuroxime	23	95.7	76.0-99.8
	Gentamicin	37	21.6	10.4-38.7
	Ciprofloxacin	43	48.8	33.6-64.3
	Levofloxacin	15	20.0	5.3-48.6
	Norfloxacin	6	83.3	36.5-99.1
	Trimethoprim/Sulfamethoxazole	41	87.8	73.0-95.4
	Erythromycin	23	52.2	31.1-72.6
	Nitrofurantoin	9	55.6	22.7-84.7
	Chloramphenicol	3	100.0	31.0-100
	Tetracycline	41	90.2	75.9-96.8
Streptococcus pyogenes	Penicillin G	1	100.0	5.5-100
	Ampicillin	9	22.2	3.9-59.8
	Ampicillin/Sulbactam	1	0.0	0.0-94.5
	Ceftriaxone	2	0.0	0.0-80.2
	Cefotaxime	12	8.3	0.4-40.2
	Erythromycin	1	100.0	5.5-100
	Nitrofurantoin	1	100.0	5.5-100
	Chloramphenicol	1	100.0	5.5-100
	Tetracycline	13	53.8	26.1-79.6
Staphylococcus saprophyticus	Penicillin G	6	100.0	51.7-100
	Ampicillin	6	100.0	51.7-100
	Amoxicillin/Clavulanic acid	11	100.0	67.9-100
	Cefuroxime	6	100.0	51.7-100
	Ciprofloxacin	11	54.5	24.6-81.9
	Levofloxacin	5	0.0	0.0-53.7
	Trimethoprim/Sulfamethoxazole	6	66.7	24.1-94.0
	Erythromycin	6	66.7	24.1-94.0
	Nitrofurantoin	5	100.0	46.3-100
	Tetracycline	11	72.7	39.3-92.7
Enterococcus sp.	Ampicillin	6	16.7	0.9-63.5
	Ciprofloxacin	7	42.9	11.8-79.8
	Levofloxacin	7	42.9	11.8-79.8
	Nitrofurantoin	3	0.0	0.0-69.0

	Tetracycline	6	83.3	36.5-99.1
Staphylococcus sp.	Penicillin G	5	80.0	29.9-98.9
	Ampicillin	5	100.0	46.3-100
	Amoxicillin/Clavulanic acid	6	100.0	51.7-100
	Cefuroxime	5	100.0	46.3-100
	Gentamicin	6	16.7	0.9-63.5
	Ciprofloxacin	6	50.0	13.9-86.1
	Levofloxacin	1	0.0	0.0-94.5
	Norfloxacin	1	0.0	0.0-94.5
	Trimethoprim/Sulfamethoxazole	5	80.0	29.9-98.9
	Erythromycin	5	80.0	29.9-98.9
	Nitrofurantoin	1	100.0	5.5-100
	Chloramphenicol	1	0.0	0.0-94.5
	Tetracycline	6	66.7	24.1-94.0
Gardnerella vaginalis	Ampicillin	3	66.6	12.5-98.2
	Ceftriaxone	2	0	0.0-80.2
	Cefotaxime	2	0	0.0-80.2
	Amikacin	1	0	0.0-94.5
	Gentamicin	3	0	0.0-69.0
	Ciprofloxacin	3	0	0.0-69.0
	Levofloxacin	3	0	0.0-69.0
	Trimethoprim/Sulfamethoxazole	3	66.6	12.5-98.2
	Tetracycline	2	100	19.8-100
Staphylococcus, coagulase negative	Piperacillin	2	100.0	19.8-100
	Amoxicillin/Clavulanic acid	2	100.0	19.8-100
	Ceftriaxone	2	100.0	19.8-100
	Amikacin	2	0.0	0.0-80.2
	Gentamicin	1	0.0	0.0-94.5
	Nalidixic acid	2	100.0	19.8-100
	Ciprofloxacin	2	100.0	19.8-100
	Levofloxacin	1	0.0	0.0-94.5
	Norfloxacin	2	100.0	19.8-100
	Nitrofurantoin	2	100.0	19.8-100
	Tetracycline	2	100.0	19.8-100
Streptococcus viridans, alpha-hem.	Cefotaxime	1	100.0	5.5-100
	Levofloxacin	1	100.0	5.5-100
Streptococcus sp.	Ampicillin	2	0.0	0.0-80.2
	Ceftriaxone	1	0.0	0.0-94.5
	Levofloxacin	1	0.0	0.0-94.5

	Tetracycline	1	100.0	5.5-100
Enterococcus faecalis	Ampicillin	1	100.0	5.5-100
	Ciprofloxacin	2	0.0	0.0-80.2
	Levofloxacin	1	0.0	0.0-94.5
	Nitrofurantoin	1	100.0	5.5-100
	Tetracycline	2	100.0	19.8-100

Table S2: Public health alerts - Important species and resistance of isolates (WHO Global Priority List of Antibiotic-Resistant Bacteria)

**Organisms**	**Alert**	**Number of isolates**	**Priority**
Enterobacterales	Carbapenems = Non-susceptible	19	High priority
Staphylococcus sp.	Vancomycin or Teicoplanin = Intermediate	1	High priority
Staphylococcus sp.	Vancomycin or Teicoplanin = Non-susceptible	14	High priority
Streptococcus, beta-hemolytic	Cephalosporin III = Non-susceptible	1	High priority
Streptococcus, beta-hemolytic	Penicillins = Non-susceptible	4	High priority
Enterobacterales	Amikacin = Non-susceptible	8	Medium priority
Enterobacterales	Possible ESBL-producing Enterobacterales	68	Medium priority
Enterococcus sp.	Vancomycin-resistant Enterococcus	1	Medium priority
Staphylococcus sp.	Vancomycin or Teicoplanin = Non-susceptible (Disk diffusion)	14	Medium priority
Streptococcus viridans	Penicillin or Ampicillin = Non-susceptible	1	Medium priority
Neisseria gonorrhoeae	Important species	3	High priority
Salmonella Typhi	Important species	2	High priority
Salmonella sp.	Important species	2	Medium priority
All organisms	Penicillins and β -lactam+Inhibitor = Discordant results	5	Medium priority
Enterobacterales	Aminoglycosides = Discordant results	2	Medium priority
Enterobacterales	Cephems = Discordant results	1	Medium priority
Enterococcus faecalis	Penicillins = Non-susceptible	2	Medium priority
Pseudomonas aeruginosa	Important species	5	Medium priority
Streptococcus sp.	Important species	2	Medium priority
